# Exploring stress response’s role in executive function impairments among adults with early adverse childhood experiences

**DOI:** 10.1038/s41598-024-53819-1

**Published:** 2024-02-19

**Authors:** Taïna Steevine Victor, Baptiste Jacquet, Farid El Massioui

**Affiliations:** 1 Université Paris 8, UFR Psychologie, 93200 Saint-Denis, France; 2 Laboratoire Cognition Humaine et Artificielle (CHArt, RNSR 200515259U), 93322 Aubervilliers, France

**Keywords:** Psychology, Risk factors

## Abstract

Adverse childhood experiences (ACEs) are recognised as precursors to numerous physical and mental health challenges. However, research on their impact on inhibitory control and working memory, particularly among healthy young adults, remains limited. The role played by the stress response as a moderator in these effects is likewise underexplored. Our study addresses this gap by examining cognitive impairments in non-clinical adults with early childhood trauma, specifically trauma before the age of 13 years, and by assessing the influence of the stress response on these effects. A total of 15 participants with early ACEs were compared with a control group (n = 18) using the Corsi Block Tapping Test (CBTT) and Stroop Word Colour Test (SCWT). Results showed that participants with early ACEs exhibited lower scores on the SCWT but not the CBTT. The stress response emerged as a potential factor in the relationship between early ACEs and cognitive performance. The implications of these findings are then discussed in relation to the existing literature.

## Introduction

Puberty is the developmental stage during which the genital organs begin to mature, and the secondary sex characteristics start to manifest. This phase typically first occurs in middle childhood, spanning from approximately 6 to 12 years of age^1–3^. The adaptive calibration model (ACM) is the theory that individuals adjust their physiological and psychological responses to chronic stress based on their early life experiences and environmental cues^1^. According to the ACM, when individuals experience severe or chronic stress during critical developmental periods such as puberty, pre and early post natal development and juvenile transition, it can have long lasting impacts on learning, growth, skills and social competition. However, puberty in the ACM compared to other stage of development is considered as highly critical^1^. Exposure to chronic stress during this period can lead to long term alterations of the stress response system (SRS)^1,3^.

The SRS initiates and inhibits physiological, psychological and behavioural responses to stress^1,4^, and throughout life, it also regulates hormone levels and the body’s physiological needs. However, in the presence of stress, there is competition for resources within the SRS, and resources that should be allocated to growth and development are instead diverted to the stress response. From the perspective of the ACM, the SRS changes to meet the demands of the immediate environment, thus adjusting to ensure its own survival^1,3,5–8^. Research has provided evidence of these changes. For example, children and adolescents who are exposed to danger have a highly sensitive amygdala as well as a reduced prefrontal cortex (PFC) and hippocampus^9^. Adults with a history of childhood abuse also have alterations in the amygdala, hippocampus and PFC^10,11^. A meta-analysis of 38 studies demonstrated that healthy adults who endured traumatic experiences in childhood exhibit reduced grey matter volume in the ventromedial PFC and anterior cingulate^12^. The aim of this paper is to explore the impact of these changes in the SRS on cognitive functions.

The SRS includes three main brain structures that coordinate its activation and regulation: PFC, amygdala and hippocampus^13^. The amygdala, hippocampus and PFC do not develop at the same rhythm. Whereas the amygdala and hippocampus mature when individuals reach middle childhood, the PFC only begins its maturation process at this time, culminating during early adulthood^2,14,15^. Thus, repeated exposure to stress during middle childhood is particularly harmful to the PFC^2^.

Originally defined in the late 1990s, adverse childhood experiences (ACEs) include a set of 10 traumatic experiences occurring in childhood^16^. To this day, ACEs are one of the most frequently referenced definitions used worldwide to assess the effects of traumatic or stressful events during childhood on the physical and mental well-being of adults^17,18^. ACEs encompass emotional, physical or sexual abuse as well as neglect and adverse upbringing, including exposure to domestic violence or being raised by a caregiver with severe mental illness or substance abuse issues^16^. Systematic reviews have consistently concluded that the amygdala, hippocampus, and PFC are the brain regions most likely to be affected by ACEs, both structurally and functionally^11,12,19,20^. One of the most robust effects of ACEs on brain structures appears to be the reduction in grey matter volume in the PFC and the hippocampus^12^.

The study of Raymond et al. has demonstrated that adults exposed to ACEs after the age of 8 exhibit attentional bias towards threat stimuli compared to those exposed earlier than 8 years old^21^. Based on the predictions of developmental stress theories, the authors proposed that exposure to adversity during puberty may have disrupted the concurrent development of prefrontal-limbic circuitry, resulting in an attentional bias towards threat^21^. Since the ACM predicts a greater impact of chronic stress on the developing PFC during puberty, this paper aims to examine how the chronic experience of stress during puberty affects cognitive processes supported by the PFC. Further research on healthy adults has provided evidence of the impact of ACEs on both the volume and activity of the PFC^10,22,23^. If a structure such as the PFC consistently exhibits these alterations, it raises questions about how the cognitive processes mediated by the PFC behave. There is indeed growing interest in exploring the long-lasting effects of repeated stressful events such as ACEs on cognitive processes mediated by the PFC.

Along with other brain regions, the PFC plays a crucial role in carrying out executive functions (EFs)^24–27^. EFs are involved in various goal-directed behaviours such as planning, decision-making, self-monitoring and self-regulation. EFs refer to a range of distinct but related cognitive processes such as inhibitory control, problem solving, reasoning, mental flexibility and working memory^24,25,27^. As an example, let us consider early adulthood when an individual’s pursuit of a successful career requires inhibitory control, which allows them to prioritise skill development or education over immediate gratification, leading to more significant long-term career achievements. EFs are thus pivotal for fostering, among others, a sense of well-being, self-fulfilment, personal development, academic accomplishments and positive interpersonal relationships^24^.

Most models use working memory, inhibitory control and cognitive flexibility as the core concepts of EFs^24–27^. Working memory is both the ability to retain and update information and to use it mentally, whereas inhibitory control is the ability to “control one’s attention, behaviour, thoughts and/or emotion to override a strong internal predisposition or external lure”^27^. Cognitive flexibility is the combination of working memory and inhibitory control and refers to the ability to change perspective^27^. In this article, we will exclusively focus on working memory and inhibitory control to investigate EFs, as they are believed to be indicative of cognitive flexibility as well^27,28^. At this stage, we would anticipate that the changes observed in the PFC of healthy adults who were exposed to ACEs would be linked to impairments in EFs among these individuals.

To date, studies have shown that the effects of ACEs persist into adulthood, impacting EFs long after the adverse events. When focusing on working memory and inhibitory control, several comprehensive reviews have revealed that individuals with mental illness such as post-traumatic stress disorder, psychosis, substance abuse disorder, bipolar disorder, schizophrenia or major depressive disorders have impairments associated with their history of ACEs^29–32^. However, mental illness can also be associated with cognitive deficits caused by the disorder itself or resulting from medication intake^13^. To mitigate the potential confounding effects of mental illness, it is thus important to consider the impact of ACEs on EFs in healthy adults. However, when focusing on working memory and inhibitory control, there are discrepancies in the findings reported in non-clinical samples of young adults under 35 years, primarily stemming from the scarcity of research in this domain^30–32^. Consequently, it is still unclear how ACEs impact EFs in young adulthood.

Focusing on the studies carried out with non-clinical samples of young adults, the results concerning working memory impairment are generally consistent. One study involving young female adults reporting a history of physical and/or sexual abuse as indicated by the Stressful Life Events Screening Questionnaire, yielded unexpected findings in terms of working memory performance based on the Spatial Emotional Match to Sample task^33^. This study revealed that individuals in the trauma group exhibited poorer working memory compared with the control group, especially in the tasks involving positive faces and challenging tests^33^. No significant between-group differences were observed for the negative and neutral tasks. Another study revealed that young adults with a history of childhood abuse (physical, sexual or psychological) exhibited lower performances compared with non-abused participants in the Operation Span task^34^. Two recent studies identified an association between childhood trauma in young adults and poorer performances in visual and verbal working memory tasks. The first study used the self-administered Childhood Trauma Questionnaire (CTQ) to evaluate past adversity. The findings indicated that higher childhood trauma scores were associated with poorer working memory^35^. The second study used the self-administered ACE questionnaire, demonstrating that neglect was linked to decreased working memory performance on the digit span task of the Weschler Adult Intelligence Scale IV^36^. However, not all studies support this association between working memory and ACEs. Using the spatial span task of the Cambridge Neuropsychological Test Automated Battery (CANTAB), the study of Li et al. found no evidence of impaired working memory in a healthy group of young adults exposed to multiple victimisation compared with a non-victimised group^37^.

Regarding inhibitory control, the studies conducted on healthy young adults who experienced childhood trauma are less consistent: some failed to provide evidence of inhibitory impairments, while others confirmed their presence. For example, using the Child Abuse Survey, Patriquin et al. revealed that young women with a self-reported history of sexual abuse before the age of 14 had comparable performances with a control group on the emotional version of the Stroop Colour Word Test (e-SCWT)^38^. Using the standard version of the SCWT, Lu et al. found no evidence of inhibitory control impairments^23^, as participants meeting the criteria for childhood trauma performed comparably to the control group. One study conducted with the Traumatic Antecedent Questionnaire showed that women who experienced sexual abuse before the age of 12 had similar performances to healthy controls on the Go/No-Go task^39^. This was confirmed by another study using the same Go/No-Go task, which showed that participants with low CTQ scores had similar performances to those with higher CTQ scores^40^. Previous research also found no evidence of inhibitory control impairment among young adults who experienced multiple victimisation compared with their non-victimised counterparts^37^. Recent studies using an emotional version of the Go/No-Go task found no evidence of inhibitory control impairments among young women with history of trauma based on the Early Trauma Inventory to assess physical, emotional and sexual abuse as well as general traumatic experiences^41^. In contrast to these findings, two studies demonstrated variations in inhibitory control among healthy young adults with a history of childhood trauma^22,42^. The findings of Daly et al. showed that the severity of childhood trauma was associated with poorer inhibitory control^42^. In the second study, which used a longitudinal sample with documented trauma histories, Demers et al. investigated the impact of trauma on an emotional version of the Go/No-Go task^22^. Surprisingly, the trauma group outperformed the control group in the negative condition, while the performances remained comparable in the neutral condition. To conclude, a consistent pattern emerges in research on the working memory of non-clinical young adults, indicating impairments among individuals with a history of childhood trauma. In terms of inhibitory control in similar samples, the findings are more varied and less conclusive. Nevertheless, regarding the various tools used to assess ACEs, very few of the aforementioned studies documented the period or onset of the adversity among the participants^22,33,36,37,39^. Only the study of Cromheeke et al. investigated how the characteristics of the abuse (age of onset, duration, frequency) impacted the impairments, with their findings revealing a significant yet small effect of the abuse characteristics on working memory impairments^33^. Further studies are required to explore how EFs behave following exposure to stress during specific developmental periods.

Furthermore, both the SCWT and emotional content in the tasks were shown to be effective at inducing stress in a controlled laboratory setting^43^. In terms of methodology, some of the studies used some form of stress-inducing material^22,23,33,38,41,42^, although none of them specifically investigated the potential mediating or moderating role of the activation of the SRS during the task.

The PFC is highly sensitive to the activity of the entire SRS. It is well established that acute stress induces neurochemical changes in the PFC, disrupts the PFC network connections and thus affects EFs^6,44,45^. In healthy adults, childhood trauma is associated with lower PFC connectivity when performing inhibitory control and working memory tasks^22,23^. It is therefore reasonable to assume that acute stress would exacerbates the potential deficits in EFs among individuals with childhood trauma.

To our knowledge, only three studies have explored the impact of acute stress on working memory or inhibitory control^41,46,47^, although none of them demonstrated how acute stress could potentially highlight discrete impairments in EFs. The first study of Jones recruited young adults with ACEs^47^. The sample was divided into two groups: one watched a video of a high school shooting (stress condition), while the other watched a video of non-aversive classroom activities (neutral condition). Before and after viewing the clips, working memory assessments were conducted using the digit span task. The findings revealed that the stress condition could not predict working memory performance. Furthermore, neither the stress condition nor the ACEs had any impact on the variation in electroencephalogram signals associated with the PFC activity^47^. In his study, Harvey adopted a slightly different approach^46^, administering the CTQ and n-Back task to a sample of young adults. The participants were randomised into two groups. The first group underwent the Trier Social Stress Test protocol, a psychosocial stressor involving public speaking and challenging arithmetic questions in a socially evaluative context (high stress condition), whereas the second group carried out a milder version of the protocol, which included speaking alone in an empty room and simple arithmetic tasks (low stress condition). Both groups performed the n-Back task immediately after the stress-inducing scenario. The findings revealed a lack of correlation between CTQ scores and working memory performances before and after the stress-inducing task. Additionally, the study provided no conclusive evidence that childhood trauma and the stress condition could predict working memory performance. However, post-hoc analysis indicated a non-significant tendency for high CTQ scores to predict greater difficulties in working memory under high stress conditions. Finally, the study of Golde et al. included a cohort of young women who had experienced multiple and severe sexual or physical trauma alongside a matched control group^41^. Both groups completed the Montreal Imaging Stress Task, a task intentionally designed to induce acute stress^43^. Before and after the task, participants’ performances on an emotional version of the Go/No-Go task were evaluated^41^. Both groups displayed greater difficulty in inhibitory control following the acute stress task, with no between-group difference. Moreover, brain imaging data revealed intriguing patterns: during the emotional Go/No-Go task in which participants had to inhibit their responses to frightening faces, those with a history of trauma had reduced PFC activation but increased anterior insula activation after the induction of stress in comparison with the healthy control group. The authors suggested that this pattern might signify the intensified allocation of neural resources in response to stress among individuals with trauma, thus causing a shift from executive control to salience detection^41^.

Aside from the use of negative affective stimuli to which individuals with childhood trauma may display reduced responsiveness^22^, another significant limitation of these studies is the sequential process of inducing stress before the evaluation of EFs. Neural activation associated with the stress response and EFs is characterised by dynamic processes. When the stress-inducing factor is eliminated during EF assessments, it is probable that the neural circuits essential for EFs stop competing for resources with the neural circuits linked to the stress response. In young adults with and without a history of childhood trauma, research indicates that during periods of stress, there is a temporary shift in the balance between the limbic regions and PFC regions^41,48^, which suggests that the acute stress response activated during the execution of tasks related to working memory and inhibitory control could impact the performance of adults with a history of childhood trauma. Previous research has identified variations in working memory among young adults with a history of childhood trauma. However, limited attention has been given to exploring the potential influence of the stress response on the observed differences and how the timing of the experienced trauma might be linked to these difficulties. Similarly, studies have revealed disparities in inhibitory control, with conflicting results in the literature. Nevertheless, the potential influence of the stress response and the role of trauma timing have not been thoroughly examined to date. The objective of our paper is therefore to fill this research gap.

Based on the insights from the ACM and the idea that intense or prolonged stress like ACEs during crucial developmental stages such as puberty may potentially impact the SRS and consequently the PFC, it is justifiable to hypothesise that individuals who experience adversity in middle childhood may display compromised working memory and inhibitory control. Consequently, our initial hypothesis posits that repeated adversity before the age of 13 is linked to diminished working memory and inhibitory control abilities in healthy young adults. Given the marked competition between acute stress and EFs in the neural system of adults with ACE, our second hypothesis asserts that the stress response of individuals and their history of recurrent adversity will act as substantial predictors for their performance in inhibitory control and working memory tasks.

## Results

The sample’s characteristics are provided in Table 1. A summary of the comparisons made between the models is provided in Tables 2, 3 and 4. In our analysis, we compared multiple models using different criteria, including Akaike information criterion (AIC), Bayesian information criterion (BIC) and Bayes Factor (BF). The AIC and BIC values provide insights into the goodness-of-fit and complexity of the models. Lower AIC and BIC values indicate a better balance between model fit and complexity, with a preference for the model with the lowest values. The BF provides a ratio of the likelihood of obtaining data (D) under the alternative (H1) and null hypothesis (H0).

1 $$\begin{aligned} BF_{1,0} = \frac{P(D|H1)}{P(D|H0)} \end{aligned}$$

**Table 1:** Samples characteristics: demographics and descriptive statistics.

Variables	Early ACE group (N=15)		Control group (N=18)	
	M	SD	M	SD
Age (years)	28.26	5.36	25.27	4.93
Sex (male/female)	(5/10)		(5/13)	
Education (levels 1–7)	6.000	1.000	5.44	1.042
Intake of contraceptive pills (yes)	(3)		(1)	
Cycle phase (menstration/follicular/luteal)	(1/3/4)		(2/4/7)	
Smoking habits (tobacco/day)	1.200	2.313	0.263	0.278
Intake of alcohol (24 h prior to test yes)	(1)		(0)	
Physical activity (2 h prior to test yes)	(2)		(2)	
Meal intake (2 h prior to test yes)	(5)		(2)	
CISS-TASK	59.733	5.861	54.722	8.864
CISS-EMOTION	46.867	14.599	47.222	12.554
CISS-avoidance	40.400	13.217	42.778	11.594
CISS-distraction	20.000	7.512	21.278	7.442
CISS-social-diversion	13.200	5.335	13.889	5.189
RS-flexibility	6.467	1.125	5.889	1.132
RS-self efficacy	8.533	1.959	7.389	1.975
RS-emotion regulation	2.667	1.047	2.056	0.938
RS-optimism	8.133	2.825	7.389	1.539
RS-cognitive focus/maintaining attention under stress	2.467	1.125	2.556	0.856
RS-score-total	28.267	6.386	25.278	4.496
STAI-Y (A) T0	37.00	8.460	32.389	9.312
STAI-Y (A) T5	36.933	9.246	31.222	5.342
STAI-Y (B)	44.067	13.693	41.611	9.037
VAS (T0)	30.800	25.149	19.222	19.658
VAS (T2)	33.733	22.998	25.556	18.302
VAS (T5)	34.133	23.769	21.944	18.386
VAS (T6)	29.571	24.911	18.529	18.497
ACE scale (personal events)				
Emotional abuse	(8)		(2)	
Physical abuse	(7)		(1)	
Sexual abuse	(2)		(1)	
Emotional neglect	(9)		(2)	
Physical neglect	(2)		(0)	
ACE scale (household dysfunction)				
Divorce	(4)		(4)	
Mother treated violently	(3)		(0)	
Substance abuse	(3)		(3)	
Mental illness	(5)		(1)	
Incarcerated relative	(0)		(0)	
Total ACE scores	2.867	1.995	0.778	1.166
SCWT (P3) no interference task	52.200	6.431	46.888	8.096
SCWT (P4) high interference task	10.533	7.747	17.610	6.980
SCWT (P5) moderate interference task	21.333	12.567	22.887	10.564
CBTT span	6.2	1.082	6.777	1.262
CBTT total scores	59.466	21.672	69.222	23.598

### Inhibitory control

Among the models tested, Model 1A (Interference task) incorporated three levels of interference in the SCWT (no interference, moderate interference and high interference). A summary of the comparison made between the models is provided in Table 2. Model 1A (Interference task) was predictive of performance in inhibitory control ($BF_{1A,0A} = 4.95621 \times 10^{28}$, AIC = 713.276, BIC = 726.251). However, the optimal model for the dataset was identified as Model 3A (Interference task * Groups), which is the interaction between the Interference task and the group category (early ACEs versus control) as predictors of inhibitory control performance ($BF_{3A,0A} = 5.465783 \times 10^{30}$, AIC = 696.084, BIC = 716.845). In this context, the early ACE group demonstrated impaired inhibitory control, as indicated by the lower scores compared with the control group, specifically in the high interference task (10.5, $SD=7.75$ vs 17.6, $SD=6.98$). In the no interference task, the early ACE group seems to have performed better than the control group but the effect size was too small to be significant in this analysis (52.2, $SD=6.43$ vs 46.9, $SD=8.10$). Focusing on the high interference task, model 2B (Groups), which is the group category (early ACEs versus control), was the best model to predict performances in inhibitory control ($BF_{2B,0B} = 6.519$, AIC = 229.118, BIC = 233.607), see Table 3. However, two models were slightly predictive of inhibitory control in this particular task: Model 7B (Groups * Heart rate), which is the interaction between the group category and the heart rate measured during the task ($BF_{7B,2B} = 0.791$, AIC = 228.091, BIC = 234.077) and model 10B (Groups * Gender), which is the interaction between the group category and the sex of participants ($BF_{10B,2B} = 0.903$, AIC = 227.824, BIC = 233.810). Participants with a higher heart rate in the early ACE group tended to have lower scores on the high interference task.

**Table 2:** Comparison of the models for the scores obtained on the SCWT for the tasks with no interference, high interference and moderate inteference.

Model name	AIC	BIC	$BF_{X,0A}$
Model 0A (null)	850.612	858.397	1
Model 1A (interference task)	713.276	726.251	$4.95621\times 10^{28}$
Model 2A (groups)	848.140	858.520	0.940
Model 3A (interference task * groups)	696.084	716.845	$5.465783\times 10^{30}$

**Table 3:** Comparison of the models for the scores obtained on the SCWT for the task with high interference.

Model name	AIC	BIC	$BF_{X,0B}$
Model 0B (null)	234.363	237.356	1
Model 2B (groups)	229.118	233.607	6.519
Model 4B (FreqCat1)	235.557	240.047	0.260
Model 5B (FreqCat2)	235.701	240.190	0.242
			$$BF_{X,2B}$$
Model 2B (groups)	229.118	233.607	1
Model 6B (groups * anxiety-sate)	230.068	236.054	0.294
Model 7B (groups * Heart rate)	228.091	234.077	0.791
Model 8B (groups * varHeart rate)	230.671	236.657	0.218
Model 9B (groups * subj stress)	231.036	237.022	0.181
Model 10B (groups * gender)	227.824	233.810	0.903
Model 11B (groups * age)	231.006	236.992	0.184
Model 12B (groups * education)	231.093	237.079	0.176

### Working memory

A summary of comparison between models is provided in Table 4. Regarding the performances on the Corsi Block Tapping Test (CBTT), none of the tested models were better than model 0D (null) (AIC = 303.369, BIC = 306.362). No significant relation was found between the ACEs, the total CBTT scores or any of the other variables that were explored. However, it is worth noting that model 14D (Heart rate), which is the mean heart rate measured during the CBTT, may have a minor influence on the total CBTT score according to the model’s AIC ($BF_{14D,0D} = 0.856$, AIC = 302.184, BIC = 306.673). Specifically, there was a slight association between heart rate and performances on the CBTT: participants with a higher heart rate tended to have poorer performances on the CBTT.

**Table 4:** Comparison of the models for total scores on the CBTT.

Model Name	AIC	BIC	$BF_{X,0D}$
Model 0D (null)	303.369	306.362	1
Model 2D (groups)	303.805	308.294	0.381
Model 4D (FreqCat1)	303.342	307.832	0.480
Model 5D (FreqCat2)	305.175	309.664	0.192
Model 13D (anxiety-sate)	305.331	309.821	0.177
Model 14D (heart rate)	302.184	306.673	0.856
Model 15D (varHeart rate)	304.650	309.139	0.249
Model 16D (Subj stress)	305.000	309.490	0.209
Model 17D (gender)	305.221	309.711	0.187
Model 18D (age)	303.917	308.407	0.36
Model 19D (education)	305.362	309.852	0.175

## Discussion

In recent years, there has been a growing interest in examining the impact of childhood trauma on executive functions in healthy young adults. While childhood trauma is often associated with resilience and protective factors that mitigate mental and physical health issues, researchers have started investigating its potential negative effects in non-clinical samples^49–52^. However, studies examining impairments in working memory and inhibitory control among healthy individuals with childhood trauma are limited and have produced contradictory findings. Moreover, there remains a gap in understanding how the stress response can moderate the relationship between childhood trauma and EFs.

Our objective was to investigate the connection between the timing of trauma and the challenges relating to working memory and inhibitory control in healthy young adults. We hypothesised that individuals who experienced childhood trauma before the age of 13 would have decreased abilities in both working memory and inhibitory control. However, our findings did not demonstrate a significant link between early childhood trauma and working memory assessed using the CBTT.

This result is contrary to previous findings in visuospatial working memory among healthy adults with trauma^33–35^. The CBTT is equivalent to the spatial span task of the CANTAB^53^. Our results are thus consistent with the study of Li et al. who found no signs of impaired working memory using the spatial span task of the CANTAB^37^. First of all, it is plausible that this type of task may lack the sensitivity of the operation span and n-Back tasks to detect variations among participants. Future research investigating working memory in healthy populations in the context of childhood trauma should therefore consider using alternative materials beyond the CBTT or spatial span task from the CANTAB. Secondly, within our early ACE group, because of the limited sample we were unable to further distinguish subgroups based on the timing of early ACEs, such as infancy (0–2 years old) or early childhood (3–7 years old). Exploring these subgroups could be relevant because, in addition to the PFC, the hippocampus also plays a role in working memory processes^54,55^ but develops during infancy^21^. In our early ACE group, reported adverse experiences occurred at a minimum after the age of 2 resulting maybe, in a minimum to no impact of ACEs on the hippocampus. It is thus possible that our participants had efficient functioning of the hippocampus, which resulted in equivalent working memory functioning compared to the control group. As suggested by Raymond et al.^21^, when conducting further investigations into how adversity affects cognitive processes, studies should consider the age of trauma in relation to the brain structures that might be vulnerable during the investigated period. Thirdly, we hypothesized that the stress response during the task would mediate performances in the trauma group. Although heart rate had a slight impact on CBTT performances, it was not specific to the early ACE group. This aspect of the question remains unanswered. Studies suggest that there is an interaction between stress and gender in working memory performance, with women tending to be less affected by stress in working memory tasks compared to men^56,57^. Since our sample consisted mostly of women, there might be a moderating effect of gender on working memory performances. This suggests that studies on stress response should either focus on one gender at a time or ensure that the samples are sufficiently balanced between men and women.

In relation to inhibitory control, our research reveals an important link between recurrent childhood trauma before the age of 13 and performance deficits in healthy young adults, as measured by the SCWT. In particular, participants with early ACEs underperformed in high interference tasks compared with the control group. This result diverges from the majority of studies in the field^22,23,38–41^, although it is supported by the study of Daly et al.^42^. Our approach seems important for evaluating the difficulties encountered in these populations, as it removes the affective component and reduces the delay between the stress induction and the EF task. On the one hand, the presence of emotional stimuli mitigates disparities, as trauma-exposed groups often show desensitisation to negative stimuli^22^. On the other hand, research has demonstrated that during a stressful task, adults with a history of trauma tend to activate limbic regions more than PFC regions that is involved in EFs^22,41^. The administration of the SCWT may have acted as both a stress-inducing task and a measure of inhibitory control that favoured the detection of discrete impairments. However, the measures of heart rate did not vary over time and between groups. Even though the SCWT appeared to be a reliable stress-inducing material, it did not increase heart rate among our participants. Therefore we cannot conclude that was due to the tasks or inherent to the participants. Our results are not surprising if we consider two potential explanations for these disparities. It is plausible that our sample size was not sufficiently large to detect heart rate differences accurately as observed by Beilharz et al.^58^. It is also worth noting that our sample differed from Beilharz’s study in terms of adverse experiences and mental health status. The study of Beilharz et al., reported that the trauma group had experienced three or more ACEs, whereas our participants have had fewer ACEs. Additionally, it’s noteworthy that the trauma group in their study reported poorer mental health compared to the control group. Therefore, the differences observed in heart rate can likely be attributed to variations in mental health status and numerous adverse experiences which is not the case in our study. To gain a more comprehensive understanding of this relationship, larger-scale studies are necessary.

Furthermore, our results suggested that individual differences, such as high heart rate and gender, played a role in inhibitory control performances. The study by Roos et al.^59^ on healthy young subjects, with no regard for past experiences of trauma, demonstrated that a high heart rate is associated with lower performances on an inhibitory control task after stress induction. In our results, heart rate alone was not predictive of inhibitory control, but rather the combination of the group factor and heart rate measure. This supported our second hypothesis that stress response might be a key factor in highlighting difficulties in inhibitory control among individuals who have experienced ACEs. However, gender was also sligthly a better predictor than heart rate when combined to the group factor. The study by Colzato et al.^60^ showed that healthy young women were less efficient in inhibitory control than men when they were in their follicular phases. Considering that our sample was predominantly composed of women, there might be a mediating impact of cycle phase to consider regarding inhibitory control performances in our results. Further studies might consider focusing on females who are either in their menstruation or luteal phases, as these phases seem to have no significant impact on inhibitory control.

Our research further highlights that the age at which trauma occurs plays a pivotal role in predicting performance outcomes in inhibitory control under the high interference condition. To our knowledge, this is the first study to demonstrate this relationship. Interestingly, early childhood trauma did not significantly influence scores on the moderate interference task. This finding has profound ramifications for contemporary society. It underscores the critical window of childhood where public policy interventions can make a difference, thus emphasising the fact that the repercussions of early childhood trauma continue into adulthood, particularly affecting inhibitory control abilities under difficult conditions. Additionally, when the subjects had a high heart rate in the early ACE group, they tended to have lower scores on the high interference task, which may explain why adults with ACEs may be disadvantaged in high-stress situations such as job interviews or exams when compared to their peers without ACEs^61^. Future research in this field is necessary to further investigate the difference between early and late childhood trauma and their impact on real-life situations.

Surprisingly, our study showed a tendency toward better performance for the early ACEs group than the control group on tasks that did not require inhibitory processes. The study of Moreno-López et al. found that resilient healthy adults have greater connectivity between the PFC and limbic regions such as the amygdala^51^. In the absence of interference, these predispositions might contribute to better performances on cognitive task^22^. Nevertheless, women with ACEs tend to pursue higher education qualifications^61^. Considering that two-thirds of our early ACE group consisted of women and that half of them had a Master’s degree, it is important to acknowledge the potential confounding factors of sex and education level on performance. Further research might be needed to better understand the factors contributing to improved functioning in individuals with ACEs.

Our study had several limitations. First, the sample size was small, and multiple variables were combined, thus leading to an increased risk of Type 1 errors in the analysis. Despite meeting the criteria for valid predictions, additional studies are needed with larger sample sizes. Second, the lack of homogeneity in the early ACEs warrants further research to explore the different types of adversity and their relationship to EFs, as existing studies have shown that sexual abuse, for example, has a specific impact on EFs^39,62^. Finally, our study was limited by the absence of objective measures for both childhood trauma history and the mental or physical health of participants. Retrospective data such as ACEs inherently suffer from an inability to verify historical information. The accuracy and recollection of past events, particularly traumatic ones, may also be influenced by public policies that guide the detection and reporting of early adversity. Without a longitudinal approach or thorough interview process, these challenges will continue to impact the collection of verified data. The same applies to the accurate assessment of mental and physical health.

## Methods

### Participants

Based on the results of Daly et al. on the SCWT task^42^, we conducted an a priori analysis before administrating the experiment. This analysis suggested that a total sample size of 28 participants would be sufficient to detect a moderate effect. However, since we did not have information on the required sample size for the CBTT task to achieve a moderate effect size and to minimize potential sample loss, we opted to recruit at least 40 participants.

The study was conducted at the Museum of Science and Industry in Paris, France. The experiment was performed in accordance with the latest version of the Declaration of Helsinki and obtained approval from the ethics committee of the University Paris 8 (protocol number CE-P8-2022-05-11). All participants provided written informed consent. A total of 40 young adults aged 19–36 years was recruited from the pool of volunteers of the Relay of Information on Cognitive Sciences. Exclusion criteria were cardiovascular disease, diabetes, visual impairment and any past or current disease for which the person takes daily medication. In addition, the study excluded individuals with diagnosed psychiatric or neurodevelopmental disorders or who underwent psychotherapy in the past 12 months. Additionally, seven subjects were excluded due to missing cardiac measurements. The final sample therefore resulted in 33 participants, 18 in the control group ($Mage=25,27$) and 15 in the early ACE group ($Mage=28,26$). Of all the sample, 23 were women. On average, participants had 2–3 years of graduate education. To better support our hypothesis, only participants who had experienced repeated ACEs before the age of 13 years were included in the early ACE group. Since the group category was only defined after the experiments, the experimenters were blind to the possible trauma of participants at the time of the study.

### Measures

Adverse childhood experience scale^16^. In our study, we used a computerised version of the 10-item ACE scale^63–65^. Participants were required to indicate whether they had experienced specific forms of abuse or household dysfunction before the age of 18 years. Participants had to respond to a total of 10 situations. An initial set of five situations related to their personal experience of physical neglect, emotional neglect, sexual abuse, physical abuse and emotional abuse. The next five situations related to their family members or household circumstances such as parental substance abuse, mental illness of a parent, exposure to domestic violence, divorce, parental abandonment, death or imprisonment of a family member. Participants were asked to respond “yes” or “no” to each situation. For the situations to which participants responded affirmatively, additional questions were asked to gather further information such as the frequency of the experiences and the age at which the events began. Participants were later included in the early ACEs group if they reported an onset before the age of 13. Furthermore, supplementary items from the Inventory of Traumatic Events (IET) were used to account for potential sources of trauma beyond ACEs^66^. These additional items pertained to experiences involving exposure to natural disasters, intentional acts of violence, deaths and accidents.

Corsi block tapping test (CBTT)^53,67^. The CBTT is a visuospatial memory task used to assess working memory. We administered a computerised version of the CBTT, the forward version according to recent recommendations^53^. The original task involves arranging a sequence of randomly positioned cubes on a desk in a specific order. Nine cubes are placed on each side of the desk and the participant is given 10 s per trial to accurately reproduce the proposed sequence. The level of difficulty increases with the growing number of cubes. The participant must wait until the sequence is complete before providing an answer. In the case of two consecutive failures at the same level, the task ends and the Corsi span is measured. The Corsi span refers to the longest sequence correctly reproduced, with the total score being calculated by multiplying the block span (length of the longest sequence) by the number of correct trials. Typically, an average individual recalls around 5–6 cubes in the correct order. In the computerised version, squares were displayed on a black screen of 255 mm * 205 mm. Each square measured 30 mm * 30 mm. Participants received sound cue to start answering. A total of 15 sequences was administered in the absence of failure.

Stroop Task or Stroop Colour and Word Test (SCWT)^68^. The SCWT is a neuropsychological test invented by Stroop in 1935. This test measures the ability to inhibit the cognitive interference that occurs during the processing of one stimulus with two attributes in competition. This test is also applied to measure cognitive functions such as selective attention or inhibitory control^23,42,69^. We administered the SCWT to assess inhibitory control according to recent recommendations^69,70^. In the standard version of the Stroop test, the participant must read or name a colour aloud as quickly as possible. Three successive tasks are presented to the participant. Each task is made up of 100 items, i.e., 10 lines of 10 items. The participant must read or name the colours for the first row from left to right and then continue to the next row. As part of our study, we present a computerised version of the Stroop with five tasks, each presented for 45 s. The following is a description of the original tasks.

- Task M congruent: the participant must read the name of the colours written in black (Red, Green, Blue).
- Task C congruent: the participant must name the colours of the rectangles shown (red, green, blue).
- Task CM incongruent: the participant must name the colour of the text without reading the word (e.g., the word ‘Red’ written in green ink). In this condition, the difficulty in inhibiting the automatic reflex reading is called the Stroop effect.

Based on recent recommendations for the administration of the Stroop test, we added two tasks to control the semantic interference produced by the type of word presented in incongruent condition^70,71^. In the computerised version, one more colour (yellow) and two additional interference conditions were added.

- Task P1 (M) congruent: the participant must read the name of the colours printed in black (Red, Green, Blue).
- Task P2 (C) congruent: the participant must name the colours of the rectangles shown (red, green, blue).
- Task P3: the participant must name the colour without reading the word (e.g., the word ‘Red’ written in red ink).
- Task P4 (CM) incongruent: the participant must name the colour without reading the word (e.g., the word ‘Red’ written in green ink). In this condition, the difficulty in inhibiting the automatic reflex reading is called the Stroop effect.
- Task P5 incongruent: the participant must name the colour without reading the neutral word presented (e.g., the word ‘Bridge’ written in blue).

The participant had 45 s to name as many colours or read as many words as possible. We then measured the number of errors, the number of errors corrected per board and the total score out of 100 items. We adopted a scoring method based on that of Golden in 1978^72^. The number of items correctly named in 45 s in each condition is obtained (P1, P2, P3, P4, P5). The predicted P1–P2 score ($P_{P1,P2}$) is calculated using the following formula:

2 $$\begin{aligned} P_{P1,P2}=\frac{P1 \times P2}{P1+P2} \end{aligned}$$

Then the $P_{P1,P2}$ value is subtracted from the number of items correctly named in the three following conditions.

- No interference condition $IP3=P3-P_{P1,P2}$
- High interference condition $IP4=P4-P_{P1,P2}$
- Moderate interference condition $IP5=P5-P_{P1,P2}$

In a laboratory setting, the Stroop task measures selective attention or inhibitory processes and induces stress due to its high cognitive demands, the constant risk of failure and the instant negative feedback regarding performance^43,58,73^. In the oral version, the Stroop task consists of a public speaking/cognitive task, which is one of the most effective ways to elicit a stress response in participants in the laboratory^74^. The SCWT in this configuration has the potential to induce a stress response. Therefore, in the procedure, it is consistently administered after the CBTT to minimize the potential influence of stress responses on CBTT performances^75,76^.

Visual analog scale (VAS)^77^. The VAS is a single-item scale rated from 0 ‘not at all stressed’ to 100 ‘very stressed’. The participants answered the VAS multiple times during the experiment. The VAS was employed to monitor changes in the participants’ emotional state over time and to provide a subjective evaluation of their emotional state.

State-Trait Anxiety Inventory for adults (Form Y) (STAI-Y)^78^. The State-Trait Anxiety Inventory (STAI-Y) is divided into two distinct scales: STAI-Y(A) to evaluate the anxiety state and STAI-Y(B) to assess the anxiety trait^79^. STAI-Y(A) consists of 20 items aimed at assessing an individual’s current emotional state, while STAI-Y(B), also comprising 20 items, evaluates the subject’s general emotional state. In both scales, participants provide responses on a Likert-type scale to indicate the intensity of their responses. Responses are scored from 1 to 4, with lower scores corresponding to lower levels of anxiety. Total scores are calculated by summing the scores obtained for each item, resulting in a range of scores from 20 to 80 for each scale. STAI-Y(A) was used to monitor changes in participants’ emotional state over time and to provide a subjective evaluation of their emotional condition. STAI-Y(B) was used as a validation measure to ensure the absence of anxiety disorders, as these psychological conditions are linked to disruptions in the SRS^80,81^.

Physiological measure of stress response. Heart rate was assessed using a BIOPAC MP36 module, with photoplethysmography (PPG) being used to illuminate the skin and track alterations in light absorption. The PPG sensor was positioned on the non-dominant hand, specifically on the tip of the ring finger^82^.

Connor and Davidson Resilience Scale 10 items (CD-RISC 10)^83^. The CD-RISC 10 comprises 10 statements encompassing various aspects of resilience. The scale primarily assesses hardiness, with items corresponding to qualities such as adaptability (items 1 and 5), self-efficacy (items 2, 4 and 9), emotion regulation ability (item 10), optimism (items 3, 6 and 8) and cognitive focus/attention maintenance under stress (item 7). Each statement is rated on a five-point scale ranging from 0 to 4. A score of 0 indicates that the statement does not hold true at all, whereas a score of 4 indicates that the statement is true almost all the time. The cumulative score is obtained by summing up all 10 items, resulting in a possible range of scores from 0 to 40. Higher scores reflect greater resilience, whereas lower scores suggest lower levels of resilience or greater difficulties in rebounding from adversity.

Coping Inventory for Stressful Situations (CISS)^84^. The CISS is a 48-item questionnaire consisting of three distinct scales assessing task-oriented, emotion-oriented and avoidance-oriented coping. Respondents are required to indicate their agreement with each item on a Likert-type scale ranging from 1 (not at all) to 5 (very much). The cumulative score is obtained by summing the responses to all the items. The CISS and CD-RISC were administered to assess the ability of resilience and coping, respectively, to act as protective factors. Healthy subjects with past trauma tend to have high resilience and productive coping strategies such as task-oriented coping^50,52^.

### Procedure

**Figure 1:**
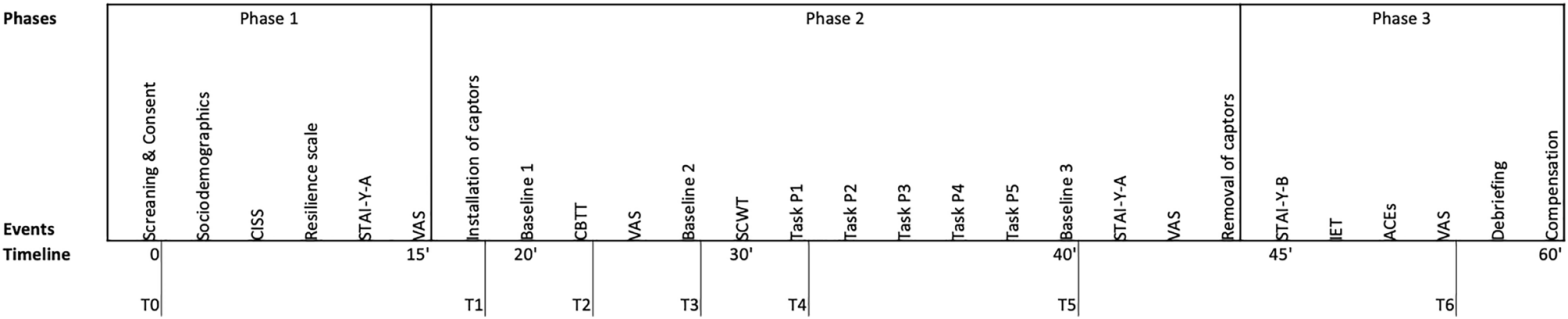
Description of the procedure. *CISS* Coping Inventory for Stressful Situations, *STAI-Y(A)* State-Trait Anxiety Inventory A form, *STAI-Y(B)* State-Trait Anxiety Inventory B form, *VAS* Visual Analog Scale, *SCWT* Stroop Colour Word Test, *IET* Inventory of Traumatic Event.

The descriptive procedure is provided in Fig. 1. Participants meeting the inclusion criteria were asked to abstain from consuming alcohol 24 h prior to the experiment and to refrain from engaging in physical exercise or eating 2 h before the experiment. In the laboratory setting, participants were informed about the study, which included answering questionnaires about their emotional state and life history events and completing two cognitive tasks. They were informed that they might experience stress during the experiment. The experiment consisted of three phases, as outlined in Fig. 1. In the first phase, participants underwent screening for sociodemographic data and exclusion criteria and completed the assessments of the CD-RISC-10, CISS, STAI-Y(A), and VAS. Following the completion of the questionnaires, participants were taken to another room to begin Phase 2.

During Phase 2, participants were seated in front of a computer screen, and the equipment for collecting electrophysiological data was set up. Cardiac frequency was measured using a PPG placed on the finger of their non-dominant hand. Participants were instructed to remain seated with minimal hand movement during Phase 2. A 2-min baseline period of rest was recorded before participants performed the first cognitive task (CBTT). Following the completion of the CBTT, participants were asked to provide a second rating on the VAS followed by another 2-min baseline period of rest. After the second baseline, participants completed the second cognitive task (SCWT). Subsequently, participants rested for 2 min before providing a second assessment of the STAI-Y(A) and a third rating on the VAS. After completing the questionnaires, participants returned to the initial room. They were offered water and sweets after Phase 2. In Phase 3, participants completed the final questionnaires, namely the STAI-Y(B), ACE scale, and complementary items of the IET followed by a fourth rating on the VAS. Lastly, participants were debriefed and received a compensation gift card valued at €10.

### Data analysis

BF was computed with the BIC and AIC values using Gaussian models and mixed Gaussian models for the repeated measures of interference. The AIC and BIC values provided insights into the goodness-of-fit and complexity of the models. Lower AIC and BIC values indicated a better balance between model fit and complexity, with the model with the lowest values being preferred.

## Data Availability

The datasets generated and analysed in the current study are provided at https://osf.io/a4pgm/?view_only=249cf9a815e44e57b2bc58e42b46d127.
